# Genetic analysis of Saker Falcon (*Falcocherrug*) subspecies

**DOI:** 10.3897/BDJ.12.e116889

**Published:** 2024-02-16

**Authors:** Rusko Petrov, Dobri Yarkov, Nayden Chakarov

**Affiliations:** 1 Green Balkans - Stara Zagora NGO, Stara Zagora, Bulgaria Green Balkans - Stara Zagora NGO Stara Zagora Bulgaria; 2 Trakia University - Stara Zagora, Stara Zagora, Bulgaria Trakia University - Stara Zagora Stara Zagora Bulgaria; 3 University of Bielefeld, Germany, Bielefeld, Germany University of Bielefeld, Germany Bielefeld Germany

**Keywords:** birds of prey, captive breeding, microsatellites, *Falcocherrugcherrug*, *Falcocherrugmilvipes*

## Abstract

Two subspecies of Saker Falcon are commonly accepted - Western (*Falcocherrugcherrug*) and Eastern (*Falcocherrugmilvipes*), which are differentiated by their distribution range and phenotype. In Bulgaria, Western Saker Falcons are breeding *ex situ* in the Wildlife Rehabilitation and Breeding Centre, part of Green Balkans - Stara Zagora NGO, with the aim of restoring the nesting population of the species in the country and both Western and Eastern - in the Breeding Centre for Birds of Prey in Burgas for the purpose of sale for the needs of falconry in the country and abroad. In 2021, a total of 115 birds from the two breeding centres were sampled. The samples were analysed in Bielefeld University (Germany) at nine microsatellite loci. Structure analyses were performed to establish the optimal explanatory number of groups. We compared the putative genetic groups with the known/expected origin of falcons. A separation in two groups best explained the allelic variation between samples. Out of 68 Saker Falcons with putatively Eastern origin, 66 were ascribed to genetic group 2 and two falcons had unclear, mixed or hybrid genetic fingerprints. Out of 42 Sakers with putatively Western origin, 33 were ascribed to genetic group 1, seven to genetic group 2 and two individuals appeared to have a mixed signature of genetic groups 1 and 2 with dominating alleles of group 2. Five known hybrids were scored as mixed signature with dominating genetic cluster 2. This suggests that the two (Eastern and Western) populations of Saker Falcon origin suggested by the subspecies' definitions are also adequate to be considered in breeding programmes. Genetic cluster 1 might represent the ancestral alleles shared with other falcons, while specific novel alleles allow the discrimination of secured Eastern Sakers (group 2), while these populations may be occasionally invaded by individuals from the west.

## Introduction

Since the first taxonomic description of the falcons in 1834 by British zoologist John Edward Gray, various scientists have defined a different number of Saker Falcon (*Falcocherrug*) subspecies (Ragyov et al. 2009), reaching up to 11 (Karyakin 2011). In the west, the popular was Charles Vaurie’s division into two subspecies - Western Saker Falcon (*Falcocherrugcherrug*) and Eastern Saker Falcon (*Falcocherrugmilvipes*) (Del Hoyo et al. 1992, Ragyov et al. 2009). However, in the latest version of the Clements Checklist of Birds of the World, there are four subspecies listed - *Falcocherrugcherrug*, *Falcocherrugcoatsi* (Dementiev, 1945) - added 2021, *Falcocherrughendersoni* (Hume, 1871) - added 2021 and *Falcocherrugmilvipes* (Clements et al. 2023). Nevertheless, to date, no clear genotypic differentiation between any subspecies has been conclusively confirmed through DNA analysis (Wink et al. 2004, Nittinger et al. 2007). Research by Nittinger et al. (2007) revealed that even the species from the subgenus Hierofalco - Saker, Gyrfalcon (*Falcorusticolus*), Lanner Falcon (*Falcobiarmicus*) and Laggar Falcon (*Falcojugger*) are not clearly differentiated genetically, which implies that they are an evolutionary young group separated from a common ancestor. Current morphological and genetic data show the Saker Falcon is monotypic, but polymorphic, exhibiting plumage variations from west to east, conveniently separating it into *Falcocherrugcherrug* and *Falcocherrugmilvipes* (Ferguson-Lees and Christie 2001). The Western individuals are with uniform brown dorsal plumage, they are smaller than the Eastern and are found in the western lowland areas of the Palearctic - in Europe, south-western Russia and Kazakhstan. The Eastern Saker Falcons are larger, with brown, dark brown and grey barred plumage on the back, inhabiting the eastern highlands of the Palearctic, such as south-eastern Russia, Mongolia and China (Eastham et al. 2002), which likely is the result of a local adaptation to the environmental conditions.

Despite the lack of evidence for subspecies differentiation, the phenotypic diversity of a species arguably ought to be protected in any case (Nittinger et al. 2007). However, further molecular investigations, genetic monitoring and establishing more molecular markers have been proposed to assist in the practical conservation of the species (Nittinger et al. 2007, Pomichal et al. 2014). In Bulgaria, Western Saker Falcons are breeding in captivity in the Wildlife Rehabilitation and Breeding Centre (WRBC), part of Green Balkans - Stara Zagora NGO and their offspring are being released with the aim of restoring the breeding population of the species in the country (Dixon et al. 2020, Lazarova et al. 2021, Petrov et al. 2021). Both Western and Eastern-type individuals are found in the Breeding Centre for Birds of Prey (BCBP) in Burgas, Bulgaria - breeding in captivity for the demands of falconry in the country and abroad. A comparative study by Petrov et al. (2023) of blood biochemical parameters of the Saker falcons from the two centres did not find conclusive differences between the putative subspecies. The goal of the present study was to examine through microsatellite analysis the potential genetic clustering of the two groups - putative Western and putative Eastern Saker Falcons. Thereby, we want to expand the understanding of the species’ genetics and aid conservation efforts on Saker Falcons and *ex situ* breeding experts in particular.

## Material and methods

In 2021, we sampled 115 falcons - 68 putatively Eastern Saker Falcons, 42 putatively Western and five known hybrids. All birds from the two facilities - WRBC and BCBP, were examined by a veterinary physician upon blood collection and were determined to be clinically healthy. We collected 0.1 ml of whole blood from either left or right basilic vein (*Vena cutanea ulnaris superficialis*) of all specimens tested. We immediately placed the blood into Eppendorf collection tubes of 1.5 ml volume containing 1 ml 90% alcohol. We used 3 ml syringes with 23G needles.

In 2022, these 115 samples of Saker Falcons were analysed at Bielefeld University, Germany at nine microsatellite loci - SSR11, SSR15, SSR45, SSR48, SSR53, SSR57, SSR63, SSR82 (Hou et al. 2018) and Fp92 (Nesje et al. 2001). Microsatellite genotyping was performed as described by Chakarov et al. (2013). M13-tagged primers for the corresponding loci were used in a 10 μl polymerase chain reaction (PCR) volume with 20–200 ng DNA, which was amplified for 35 cycles using a Type-it microsatellite PCR kit (QIAGEN), following the manufacturer's standard protocols and using an annealing temperature of 56°C. Diluted amplification products (1 μl; 1:20 dilution) were then resolved on an ABI 3730 Automated DNA Analyser (Applied Biosystems). Fragment lengths were scored for all individuals using Genemarker 1.95 (SoftGenetics LCC). Structure analyses (Pritchard et al. 2000, Falush et al. 2007) were performed for K = 1 to K = 7 for 300,000 generations with burn-in of 50,000 generations. Structure Harvester was used to establish the optimal explanatory number of groups by implementing the Evanno method (Earl and vonHoldt 2011).

## Results

**Figure 1.**
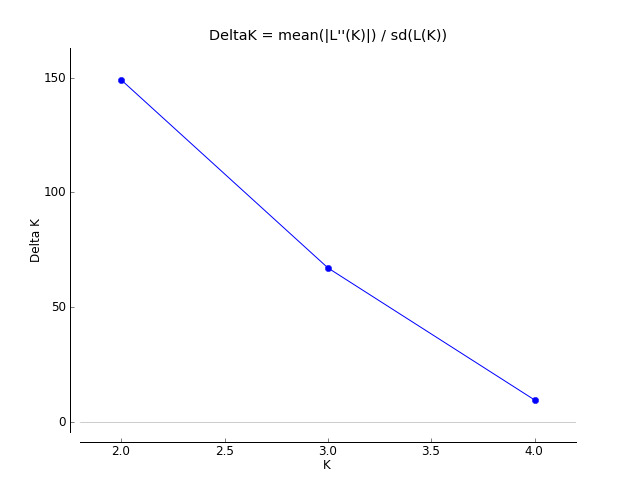
Indication for the existence of two separate Saker Falcon clusters.

**Figure 2.**
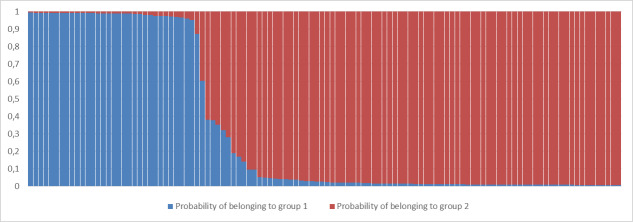
Proportions of membership of individual Saker Falcons to each of the two inferred clusters.

We compared the putative genetic groups with the known or expected origin of the falcons from the breeding facilities - the ones with incomplete pedigree information had been assigned to a group (Western or Eastern), based on history and appearance. Structure Harvester indicated that separation in two genetic clusters best explains the allelic variation between samples (Figs 1, 2). A peak at K = 2 at the ΔK graph indicates the existence of two populations which was observed in all independent simulations (10 runs).

Out of 68 Sakers with Eastern origin, 66 were ascribed to genetic group 2, two appeared to have mixed genetic signatures. Out of 42 Sakers with putatively Western origin, 33 were ascribed to genetic group 1, seven to genetic group 2 and two individuals appeared to have mixed signals from genetic groups 1 and 2 with a dominating signature of group 2. Five known hybrids (incl. one Lanner x Gyrfalcon - *Falcobiarmicus* x *Falcorusticolus*) were scored as mixes with dominating genetic cluster 2 (Table 1).

## Discussion

We found a segregation of Saker Falcon microsatellite alleles into two genetic clusters. This corresponds both to the expected and conservatively described separation of the species into two main subspecies, as well as to the putative origin/heritage of most of the sampled birds which have been bred in captivity over several generations.

Our results, showing two genetic clusters of Sakers, resonate both with morphology-based literature and subspecies descriptions. Importantly, almost 80% of the putatively Western-origin falcons were ascribed to a separate cluster, while more than 95% of the putative Eastern-originating birds segregated into another cluster. The latter cluster also included hybrids and a hybrid of other hierofalcons. This may indicate that this western cluster includes more ancestral alleles which are also shared with related species, possibly through interbreeding. In contrast, the eastern subspecies appears to be a branch with more derived alleles with possibly more recent origination. This hypothesis is in line with the “out of Africa” origin that has been suggested by other authors (Nittinger et al. 2005, Nittinger et al. 2007, Pan et al. 2017, Hu et al. 2022, Zinevich et al. 2023), which follows the migration of hierofalcons from Africa through Europe to Asia. Nevertheless, 15% of the tested birds with suspected western origin were ascribed to group 2, i.e. Eastern genotypes. This could be indicating that these birds’ genealogy was not correctly tracked during generations of breeding in captivity. Alternatively, eastern-type alleles may be represented in the western populations due to the incomplete lineage sorting of a relatively new species as the hierofalcons are (Nittinger et al. 2007).

Importantly, our findings should not be interpreted as a lack of evidence for the existence of another two separate subspecies - *F.c.coatsi*, found in the plains of Transcaspia to eastern Uzbekistan and southern Kazakhstan and *F.c.hendersoni*, found in the Pamir Mountains east to the Tibetan Plateau, as described in the latest version of the Clements Checklist of Birds of the World (Clements et al. 2023). We did not sample and analyse individuals specifically originating from the core distribution areas of these populations and, thus, cannot make any statement about their genetic separation. As both of these subspecies have smaller and more disjunct distributions than the two main subspecies *F.c.cherrug* and *F.c.milvipes*, specific sampling efforts will be needed to derive genetic material from such birds for analyses.

## Conclusions

In conclusion, as few as nine microsatellites were sufficient to recover the genetic segregation of Eastern and Western Saker Falcons. These two subspecies and associated morphologies should, therefore, be certainly considered during breeding programmes aimed at releasing birds into the wild in order to preserve genetic diversity without causing unnecessary interbreeding and disruption of potential locally-adapted alleles of functional genes. Thus, whenever doubts about the pedigree or origin of Saker Falcons in a breeding stock exist, these should be clarified through additional genetic fingerprinting.

## Figures and Tables

**Table 1.** Membership of the sampled falcons to a genetic group.

	n	Genetic cluster 1	Genetic cluster 2	Mixed genetic signature	Mixed, dominating signature of cluster 2
Putative Western Saker Falcons	42	33	7	0	2
Putative Eastern Saker Falcons	68	0	66	2	0
Known hybrids	5	0	0	0	5

## References

[B10920082] Chakarov Nayden, Jonker Rudy M., Boerner Martina, Hoffman Joseph I., Krüger Oliver (2013). Variation at phenological candidate genes correlates with timing of dispersal and plumage morph in a sedentary bird of prey. Molecular Ecology.

[B10899368] Clements J. F., Rasmussen P. C., Schulenberg T. S., Iliff M. J., Fredericks T. A., Gerbracht J. A., Lepage D., Spencer A., Billerman S. M., Sullivan B. L., Wood C. L. The eBird/Clements checklist of Birds of the World: v2023. https://www.birds.cornell.edu/clementschecklist/download/.

[B10899385] Del Hoyo J., Elliott A., Sargatal J. (1992). Handbook of the birds of the world.

[B10899395] Dixon Andrew, Ragyov Dimitar, Izquierdo David, Weeks Darren, Rahman Md. Lutfor, Klisurov Ivaylo (2020). Movement and survival of captive-bred saker falcons *Falcocherrug* released by wild hacking: Implications for reintroduction management. Acta Ornithologica.

[B10899415] Earl Dent A., vonHoldt Bridgett M. (2011). Structure Harvester: a website and program for visualizing Structure output and implementing the Evanno method. Conservation Genetics Resources.

[B10899424] Eastham Chris P., Nicholls Mike K., Fox Nick C. (2002). Morphological variation of the saker (*Falcocherrug*) and the implications for conservation. Biodiversity and Conservation.

[B10899433] Falush D., Stephens M., Pritchard J. K. (2007). Inference of population structure using multilocus genotype data: dominant markers and null alleles. Molecular Ecology Notes.

[B10899442] Ferguson-Lees J., Christie D. A. (2001). Raptors of the World.

[B10899450] Hou Xian, Xu Pengwei, Lin Zhenzhen, D'urban‐Jackson Josephine, Dixon Andrew, Bold Batbayar, Xu Jiliang, Zhan Xiangjiang (2018). Integrated tool for microsatellite isolation and validation from the reference genome and their application in the study of breeding turnover in an endangered avian population. Integrative Zoology.

[B10899465] Hu Li, Long Juan, Lin Yi, Gu Zhongru, Su Han, Dong Xuemin, Lin Zhenzhen, Xiao Qian, Batbayar Nyambayar, Bold Batbayar, Deutschová Lucia, Ganusevich Sergey, Sokolov Vasiliy, Sokolov Aleksandr, Patel Hardip R., Waters Paul D., Graves Jennifer Ann Marshall, Dixon Andrew, Pan Shengkai, Zhan Xiangjiang (2022). Arctic introgression and chromatin regulation facilitated rapid Qinghai-Tibet Plateau colonization by an avian predator. Nature Communications.

[B10899492] Karyakin I. (2011). Subspecies population structure of the saker falcon range. Raptors Conservation.

[B10899501] Lazarova Ivanka, Petrov Rusko, Andonova Yana, Klisurov Ivaylo, Dixon Andrew (2021). Re-introduction of the saker falcon (*Falcocherrug*) in Bulgaria - preliminary results from the ongoing establishment phase by 2020. Biodiversity Data Journal.

[B10899511] Nesje M., Røed K. H., Lifjeld J. T., Lindberg P., Steen O. F. (2001). Genetic relationships in the peregrine falcon (*Falcoperegrinus*) analysed by microsatellite DNA markers. Molecular Ecology.

[B10899521] Nittinger F., Haring E., Pinsker W., Wink M., Gamauf A. (2005). Out of Africa? Phylogenetic relationships between *Falcobiarmicus* and the other hierofalcons (Aves: Falconidae). Journal of Zoological Systematics and Evolutionary Research.

[B10899531] Nittinger FRANZISKA, Gamauf ANITA, Pinsker WILHELM, Wink MICHAEL, Haring ELISABETH (2007). Phylogeography and population structure of the saker falcon (*Falcocherrug*) and the influence of hybridization: mitochondrial and microsatellite data. Molecular Ecology.

[B10899541] Pan Shengkai, Zhang Tongzuo, Rong Zhengqin, Hu Li, Gu Zhongru, Wu Qi, Dong Shanshan, Liu Qiong, Lin Zhenzhen, Deutschova Lucia, Li Xinhai, Dixon Andrew, Bruford Michael W., Zhan Xiangjiang (2017). Population transcriptomes reveal synergistic responses of DNA polymorphism and RNA expression to extreme environments on the Qinghai–Tibetan Plateau in a predatory bird. Molecular Ecology.

[B10899560] Petrov R., Andonova Y., Gancheva Y., Klisurov I. (2021). Implications of captive breeding for the reintroduction of the Saker falcon (*Falcocherrug*) in Bulgaria. Agricultural Science and Technology.

[B10899569] Petrov Rusko, Lazarova Ivanka, Yarkov Dobry, Andonova Yana, Dimitrova Stefka (2023). First biochemical comparison between saker falcon subspecies *Falcocherrugcherrug* and *Falcocherrugmilvipes*. Journal of Raptor Research.

[B10899579] Pomichal Krisztián, Vági Balázs, Csörgő Tibor (2014). A case study on the phylogeny and conservation of saker falcon. Ornis Hungarica.

[B10899588] Pritchard Jonathan K, Stephens Matthew, Donnelly Peter (2000). Inference of population structure using multilocus genotype data. Genetics.

[B10899611] Ragyov D., Kmetova E., Dixon A., Franz K., Koshev Y., Nedialkov N. (2009). Saker falcon, *Falcocherrug*, reintroduction in Bulgaria: Feasibility study.

[B10920099] Wink M., Sauer-Gürth H., Ellis D., Kenward R., Chancellor R. D., Meyburg B. U. (2004). Raptors worldwide.

[B10899597] Zinevich Liudmila, Prommer Mátyás, Laczkó Levente, Rozhkova Daria, Sorokin Alexander, Karyakin Igor, Bagyura János, Cserkész Tamás, Sramkó Gábor (2023). Phylogenomic insights into the polyphyletic nature of Altai falcons within eastern sakers (*Falcocherrug*) and the origins of gyrfalcons (*Falcorusticolus*). Scientific Reports.

